# Contraceptive implant migration to the lung

**DOI:** 10.1259/bjrcr.20200216

**Published:** 2021-05-12

**Authors:** Anuj Wali, Rajdeep Bilkhu, Victoria Rizzo, Andrea Bille

**Affiliations:** 1 Department of Thoracic Surgery, Guy’s Hospital, London, UK

## Abstract

A 27-year-old female presented with a ‘missing’ contraceptive implant. Chest imaging demonstrated a 4-cm linear opacity in a subsegmental branch of the pulmonary artery to the left lower lobe consistent with a migrated contraceptive implant.

A mini-thoracotomy and arteriotomy was performed. The artery was opened distally to its third division. However, it was not possible to retrieve the implant, and the decision was made to proceed to segmentectomy. After resection, it was noted that the foreign body had significantly endothelialised within the wall of the artery and required sharp dissection for removal.

This is the first case report to demonstrate the complete endothelialisation and subsequent difficulty in removal of an embolised contraceptive implant. We hope this report adds to the growing body of literature to guide management of this extremely rare but serious complication.

## Clinical presentation

A 27-year-old female (body mass index, BMI 20.2), who had been using a contraceptive implant for 8 years, came to be evaluated after feeling generally unwell and after a return of irregular menstruation. Her current etonogestrel implant (Nexplanon^®^, Merck & Co, USA) was inserted by her GP into the medial aspect of her left upper arm two years prior to presentation. The implant was placed in the same location as two previous implants, both of which were in place for three years prior to removal and replacement. At the time removal was requested, the implant was not palpable in the arm. The patient reported it last being palpable two months before presentation.

## Investigations

**Figure 1.**
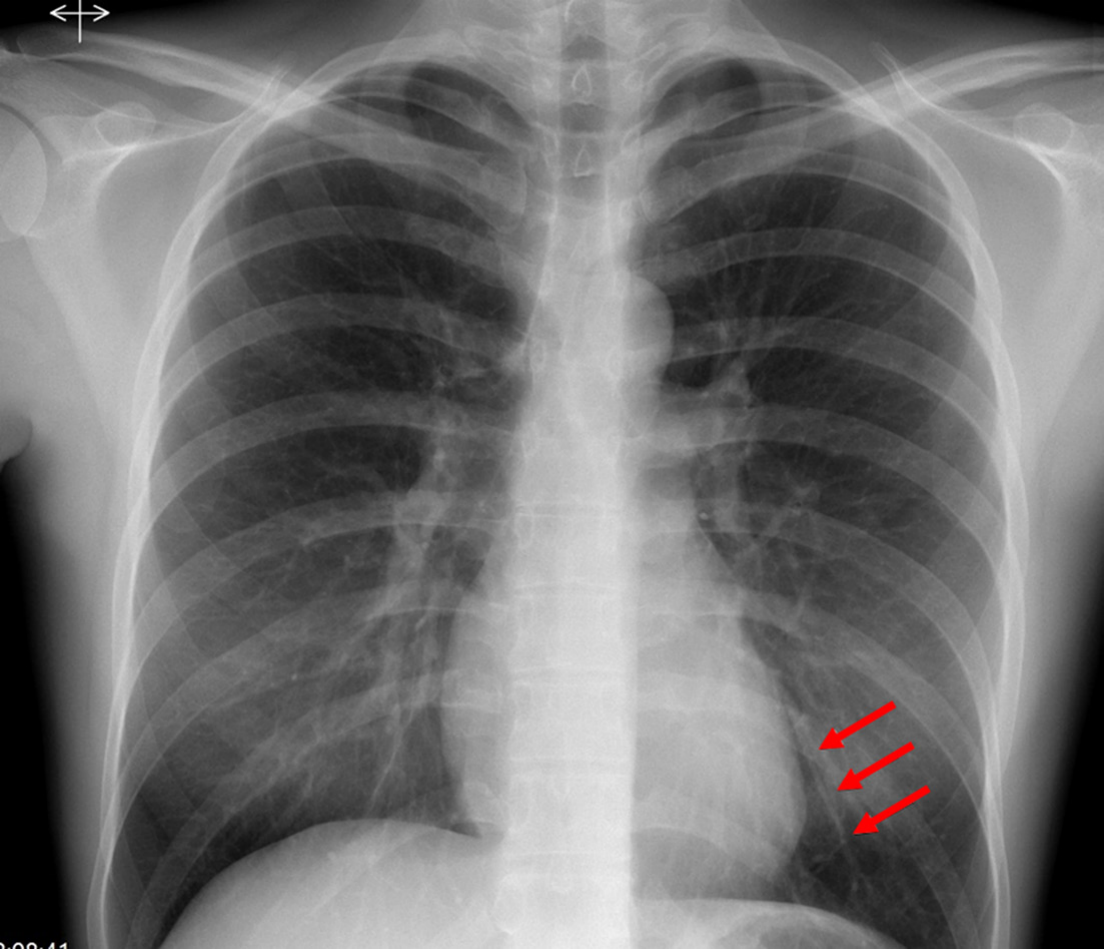
Chest X-ray with arrows demonstrating 4 cm linear opacity in the left lower zone.

**Figure 2.**
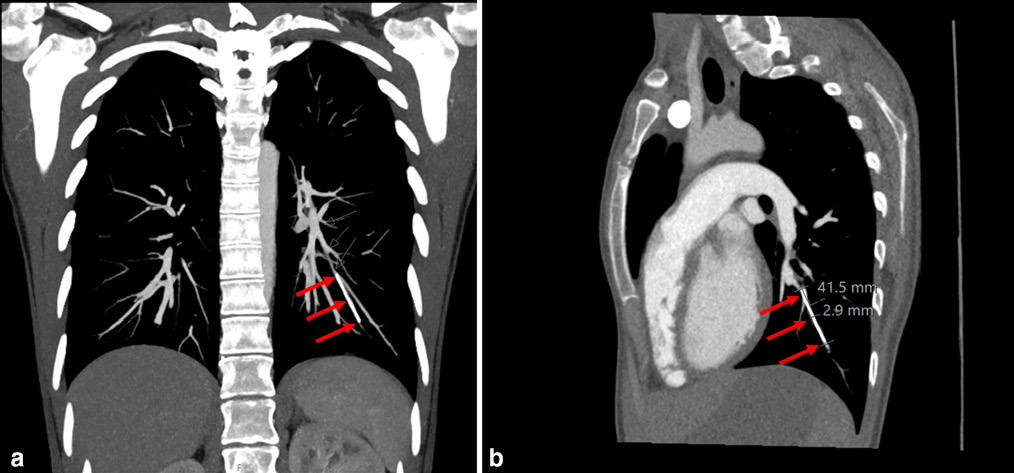
(a) Coronal section of CT pulmonary angiogram with arrows demonstrating 4 cm linear opacity in subsegmental branch of the pulmonary artery supplying the left lower lobe. (b) – Saggital section of CT pulmonary angiogram with arrows demonstrating 4 cm linear opacity in subsegmental branch of the pulmonary artery supplying the left lower lobe

The implant was not visible on X-ray or ultrasound of the left arm. She had no significant past medical history and normal lung function. The patient was referred to a specialist contraceptive tertiary unit, who performed a chest radiograph, which demonstrated a 4 cm linear opacity in the left lower zone (Figure 1). A CT pulmonary angiogram (CTPA) was performed and demonstrated a hyperdense structure within a subsegmental branch of the left pulmonary artery in the posterior basal segment consistent with the migrated contraceptive implant (Figure 2)

## Treatment

The patient was keen to have a family in the future and requested removal of the device. She was referred to Interventional Radiology and Thoracic Surgery for opinion and counselled on her options. Given the risks of not being able to retrieve the foreign body radiologically, the patient’s preference was to undergo open, lung-sparing retrieval of the foreign body with the possibility of lung resection.

A mini-thoracotomy and arteriotomy was performed to locate the foreign body. The implant was palpable within the left lower lobe. After obtaining proximal control of the pulmonary artery with an atraumatic vascular clamp, the pulmonary artery was opened. It was opened distally to its third division; however, it was not possible to retrieve the implant and so the decision was made to proceed to segmentectomy.

On removal of the resected portion of lung, it was noted that the foreign body had significantly endothelialised within the wall of the artery. Sharp dissection was required to remove the implant from the wall of the artery as it had become totally encased within endothelium (see Supplementary Video). Given the distal location and adherence of the implant to the vessel wall, radiological retrieval would not have been possible. The patient had an uneventful recovery and was discharged four days after the procedure. On review in clinic 6 weeks later, the patient had recovered well and was using alternative forms of contraception.

Supplementary Video 1.

## Discussion

This is the first reported case clearly demonstrating endothelialisation of a contraceptive implant within the wall of the pulmonary artery. This report is also the first case describing an open segmental lung resection for removal of an embolised contraceptive implant. The first reported case^1^ was in 2014, and there have been 11 further cases reported.^2^ Reported risk factors for intravascular migration of implants include low patient BMI, deep insertion and insertion near the mobile joint space. It may be possible that multiple insertions at the same site also increase the risk of migration.^3^

Once inserted, regular self-checks should be performed to ensure the implant is still palpable sub dermally. If not, there should be prompt referral from primary care to specialist centres who can perform urgent imaging to locate the device. Conservative management, Interventional Radiology, Video Assisted Thoracic Surgery and open surgical techniques have all been used to manage embolised implants.^2^ Ultimately, management of the embolised contraceptive implant depends on the location of the implant and most importantly the patient’s wishes. We recommend that minimally invasive techniques are attempted in the first instance with prompt referral to interventional radiology for attempted retrieval of an embolised device. A majority of the embolised implants reported in the literature were located in distal subsegmental branches of the pulmonary artery which presents a more technically challenging target for endovascular retrieval. Interestingly, 73% of all reported migrated implants lodged themselves within the left lower lobe lung vasculature.^2^

In our case, we hypothesise that the long latent period between implant embolisation and detection resulted in an intravascular inflammatory process that caused significant endothelialisation within the vessel wall. This means that any attempt at endovascular retrieval of this implant would likely have been futile and with increased risk of vascular injury.

Referral to thoracic surgery should be considered when an embolised foreign body has been *in situ* for an extended period, as this increases the likelihood that the device is adherent or encased within the vessel wall. This allows for comprehensive counselling from both specialties in order to offer the most pragmatic and patient focused approach to removal of the implant. In multiple previous cases reported in the literature, the migrated implants were left *in situ*, and a follow-up plan was established, consisting in regular testing of etonogestrel levels to measure its activity. There are multiple reports of failed endovascular retrieval and one case report hypothesises that this is because the Nexplanon device is designed to incite a local fibrous reaction for fixation within the soft tissues of the arm resulting in fixation within the arterial wall.^4^ The conservative approach is reasonable in the asymptomatic patient who has no desire to conceive; with the rationale being that an implant has a shelf life and will eventually become inactive.^5^ However, there is always a risk of further complication (chest pain, pneumothorax, shortness of breath etc.) when leaving a foreign body *in situ* and the patient should be adequately counselled of these risks.

We suggest that if there is a 6 week delay between implant embolisation and detection then the possibility of vascular endothelialisation and difficult removal should be considered in the treatment counselling process. Ultimately, there is still very little evidence in the literature to offer comprehensive counselling and management should be predicated by the patient’s wishes.

## Conclusion

Migration of a contraceptive implant to the lung is an extremely rare but significant complication. We report this rare case in order to highlight the risk of endothelialisation of foreign bodies that may lead to an increased risk of failed interventional procedures when attempting minimally invasive retrieval. Open thoracic surgery remains a safe and definitive approach for removal if endovascular approaches are not appropriate or unsuccessful.

## Learning points

- Clinicians should always bear in mind the possibility of distant embolisation of medical foreign bodies when referred a ‘missing device’.
- Patients should be encouraged to perform regular self-checks to monitor any implanted medical devices
- Interventional radiologists should be aware of the risks of endothelialisation of foreign bodies within vessel walls. If the foreign body has been *in situ* for a long period of time then it is important to counsel the patient on the increased risk of unsuccessful endovascular retrieval.

## References

[1] Patel A, Shetty D, Hollings N, Dodds N. Contraceptive implant embolism into the pulmonary artery. Ann Thorac Surg 2014; 97: 1452. doi: 10.1016/j.athoracsur.2013.09.029 24694432

[2] Hindy J-R, Souaid T, Larus CT, Glanville J, Aboujaoude R. Nexplanon migration into a subsegmental branch of the pulmonary artery: a case report and review of the literature. Medicine 2020; 99: e18881. doi: 10.1097/MD.0000000000018881 31977894 PMC7004701

[3] Ohannessian A, Levy A, Jaillant N, Tanguy Le Gac Y, D'Journo X, Vidal V, et al. A French survey of contraceptive implant migration to the pulmonary artery. Contraception 2019; 100: 255–7. doi: 10.1016/j.contraception.2019.05.016 31194964

[4] O' Brien A, O'Reilly MK, Sugrue G, Lawler L, Farrelly C. Subdermal contraceptive implant embolism to a pulmonary artery. Ann Thorac Surg 2015; 99: 2254–5 vol. . doi: 10.1016/j.athoracsur.2014.12.017 26046898

[5] Shekarforoush M, Chapman S, Moriarty HK, Koukounaras J, Goh GS, Clements W. Implanon NXT embolisation into the pulmonary arterial tree. Aust J Gen Pract 2020; 49: 585–6. doi: 10.31128/AJGP-12-19-5175 32864680

